# Supplementary material for: Education for all in action: Measuring teachers’ competences for inclusive education
Source: PLoS One. 2023 Nov 8;18(11):e0291033. doi: 10.1371/journal.pone.0291033 (PMC10631664; doi:10.1371/journal.pone.0291033)
Supplement: Attachment — Submitted filename: PONE-D-22-31586_Reviewer_comments.pdf [file pone.0291033.s001.pdf]

PLOS ONE

Education for all in action: Measuring teachers’ competences for inclusive education

--Manuscript Draft--

**Manuscript Number:**	PONE-D-22-31586
**Article Type:**	Research Article
**Full Title:**	Education for all in action: Measuring teachers’ competences for inclusive education
**Short Title:**	Measuring teachers’ competences for inclusive education
**Corresponding Author:**	Wendelien Vantieghem<br>Ghent University: Universiteit Gent<br>Gent, Oost-Vlaanderen BELGIUM
**Keywords:**	Inclusion; factor analysis; social attitudes; teacher attitudes; self efficacy; student diversity
**Abstract:**	<p class="Keywords">While inclusive education has achieved international importance, there is no valid instrument to measure teachers’ competences in creating quality classrooms for diverse learners, which this study aims to remedy. Explorative and confirmatory factor analyses with 975 pre-service teachers and 600 in-service teachers were used. Central to teachers’ inclusive teaching is both beliefs and efficacy. Results show that beliefs consist of four factors on student diversity, specifically ethnicity, disability, SES, and gender, as well as three factors on the responsibility of the educational field to create inclusion: general school policy, initiatives geared specifically towards ethnic minority students and towards students with a disability. Furthermore, the results show five factors related to self-efficacy: noticing student diversity, enabling high-quality student-interactions, creating stimulating learning environments, collaborating with colleagues and diverse parents. The factorial structure and scale-scores are discussed for what they unveil of teachers’ thinking about diversity in the classroom.<o:p></o:p></p>
**Order of Authors:**	Wendelien Vantieghem
	Iris Roose
	Karin Goosen
	wouter schelfhout
	Piet Vanavermaet
**Additional Information:**	
**Question**	**Response**
**Financial Disclosure**<br><br>Enter a financial disclosure statement that describes the sources of funding for the work included in this submission. Review the submission guidelines for detailed requirements. View published research articles from *PLOS ONE* for specific examples.<br><br>This statement is required for submission and **will appear in the published article** if the submission is accepted. Please make sure it is accurate.	This research did not receive any specific grant from funding agencies in the public, commercial, or not-for-profit sectors.

Powered by Editorial Manager® and ProduXion Manager® from Aries Systems Corporation

**Unfunded studies**<br>Enter: *The author(s) received no specific funding for this work.*<br><br>**Funded studies**<br>Enter a statement with the following details:<br>• Initials of the authors who received each award<br>• Grant numbers awarded to each author<br>• The full name of each funder<br>• URL of each funder website<br>• Did the sponsors or funders play any role in the study design, data collection and analysis, decision to publish, or preparation of the manuscript?<br>• **NO** - Include this sentence at the end of your statement: *The funders had no role in study design, data collection and analysis, decision to publish, or preparation of the manuscript.*<br>• **YES** - Specify the role(s) played.<br><br>* typeset	
**Competing Interests**<br><br>Use the instructions below to enter a competing interest statement for this submission. On behalf of all authors, disclose any competing interests that could be perceived to bias this work—acknowledging all financial support and any other relevant financial or non-financial competing interests.<br><br>This statement is required for submission and **will appear in the published article** if the submission is accepted. Please make sure it is accurate and that any funding sources listed in your Funding Information later in the submission form are also declared in your Financial Disclosure statement.<br><br>View published research articles from *PLOS ONE* for specific examples.	The authors have declared that no competing interests exist.

Powered by Editorial Manager® and ProduXion Manager® from Aries Systems Corporation

**NO authors have competing interests**<br><br>Enter: *The authors have declared that no competing interests exist.*<br><br>**Authors with competing interests**<br><br>Enter competing interest details beginning with this statement:<br><br>*I have read the journal's policy and the authors of this manuscript have the following competing interests: [insert competing interests here]*<br><br>* typeset	
**Ethics Statement**<br><br>Enter an ethics statement for this submission. This statement is required if the study involved:<br><br>• Human participants<br>• Human specimens or tissue<br>• Vertebrate animals or cephalopods<br>• Vertebrate embryos or tissues<br>• Field research<br><br>Write "N/A" if the submission does not require an ethics statement.<br><br>General guidance is provided below. Consult the submission guidelines for detailed instructions. **Make sure that all information entered here is included in the Methods section of the manuscript.**	Due to the anonymous and non-invasive nature of the research (being a voluntary, one-time participation of adults in survey-research on professional practice), no official approval by the ethics committee was sought. Following the European Code of Conduct for Research Integrity, all respondents agreed to a written informed consent stating that participation in the research was voluntary, that they could stop their participation at any moment, and that their answers would be processed anonymously.

Powered by Editorial Manager® and ProduXion Manager® from Aries Systems Corporation

**Format for specific study types**<br><br>**Human Subject Research (involving human participants and/or tissue)**<br>• Give the name of the institutional review board or ethics committee that approved the study<br>• Include the approval number and/or a statement indicating approval of this research<br>• Indicate the form of consent obtained (written/oral) or the reason that consent was not obtained (e.g. the data were analyzed anonymously)<br><br>**Animal Research (involving vertebrate animals, embryos or tissues)**<br>• Provide the name of the Institutional Animal Care and Use Committee (IACUC) or other relevant ethics board that reviewed the study protocol, and indicate whether they approved this research or granted a formal waiver of ethical approval<br>• Include an approval number if one was obtained<br>• If the study involved *non-human primates*, add *additional details* about animal welfare and steps taken to ameliorate suffering<br>• If anesthesia, euthanasia, or any kind of animal sacrifice is part of the study, include briefly which substances and/or methods were applied<br><br>**Field Research**<br><br>Include the following details if this study involves the collection of plant, animal, or other materials from a natural setting:<br>• Field permit number<br>• Name of the institution or relevant body that granted permission	
**Data Availability**<br><br>Authors are required to make all data underlying the findings described fully available, without restriction, and from the time of publication. PLOS allows rare exceptions to address legal and ethical concerns. See the PLOS Data Policy and FAQ for detailed information.	Yes - all data are fully available without restriction

A Data Availability Statement describing where the data can be found is required at submission. Your answers to this question constitute the Data Availability Statement and **will be published in the article**, if accepted.<br><br>**Important:** Stating 'data available on request from the author' is not sufficient. If your data are only available upon request, select 'No' for the first question and explain your exceptional situation in the text box.<br><br>Do the authors confirm that all data underlying the findings described in their manuscript are fully available without restriction?	
**Describe where the data may be found in full sentences. If you are copying our sample text, replace any instances of XXX with the appropriate details.**<br><br>• If the data are **held or will be held in a public repository**, include URLs, accession numbers or DOIs. If this information will only be available after acceptance, indicate this by ticking the box below. For example: *All XXX files are available from the XXX database (accession number(s) XXX, XXX.).*<br>• If the data are all contained **within the manuscript and/or Supporting Information files**, enter the following: *All relevant data are within the manuscript and its Supporting Information files.*<br>• If neither of these applies but you are able to provide **details of access elsewhere**, with or without limitations, please do so. For example:<br><br>*Data cannot be shared publicly because of [XXX]. Data are available from the XXX Institutional Data Access / Ethics Committee (contact via XXX) for researchers who meet the criteria for access to confidential data.*<br><br>*The data underlying the results presented in the study are available from (include the name of the third party*	After acceptance, all data files will be made available via the Zenodo database (https://zenodo.org/).

and contact information or URL).<br>• This text is appropriate if the data are owned by a third party and authors do not have permission to share the data.<br><br>* typeset	
Additional data availability information:	Tick here if the URLs/accession numbers/DOIs will be available only after acceptance of the manuscript for publication so that we can ensure their inclusion before publication.

Powered by Editorial Manager® and ProduXion Manager® from Aries Systems Corporation

Manuscript Click here to access/download;Manuscript;draft 7 - title page + doc for review.docx

Education for all in action: Measuring teachers' competences for inclusive education

Authors:
Vantieghem, Wendelien[a]; Roose, Iris[b]; Goosen, Karin[a]; Schelfhout, Wouter[c]; Van Avermaet, Piet[a]

Contact information:
[a:] Centre for Diversity & Learning, Ghent University; Sint-Pietersnieuwstraat 49, 9000 Ghent, Belgium
wendelien.vantieghem@ugent.be (corresponding author); Karin.Goosen@UGent.be; piet.vanavermaet@ugent.be
[b] Human & society, Artevelde University College, Hoogpoort 15, 9000 Ghent, Belgium
iris.roose@arteveldehs.be
[c] Antwerp School of Education, University of Antwerp, Venusstraat 35, 2000 Antwerpen, Belgium
wouter.schelfhout@uantwerpen.be

Word count
6759 words (excl. references)

Funding: This research did not receive any specific grant from funding agencies in the public, commercial, or not-for-profit sectors.

Competing interest statement: none

Education for all in action:

Measuring teachers’ competences for inclusive education

While inclusive education has achieved international importance, there is no valid instrument to measure teachers’ competences in creating quality classrooms for diverse learners, which this study aims to remedy. Explorative and confirmatory factor analyses with 975 pre-service teachers and 600 in-service teachers were used. Central to teachers’ inclusive teaching is both beliefs and efficacy. Results show that beliefs consist of four factors on student diversity, specifically ethnicity, disability, SES, and gender, as well as three factors on the responsibility of the educational field to create inclusion: general school policy, initiatives geared specifically towards ethnic minority students and towards students with a disability. Furthermore, the results show five factors related to self-efficacy: noticing student diversity, enabling high-quality student-interactions, creating stimulating learning environments, collaborating with colleagues and diverse parents. The factorial structure and scale-scores are discussed for what they unveil of teachers’ thinking about diversity in the classroom.

Keywords: inclusion; factor analysis; social attitudes; teacher attitudes; self efficacy; student diversity

Introduction

Nowadays, due to the expansion of education, migration flows, and various policy initiatives, classrooms are becoming increasingly diverse (Banks et al., 2007; Forghani-Arani et al., 2019). At the same time, inclusive education has achieved international importance as a leading educational movement (Burns & Shadoina-Gersing, 2010; Van Mieghem et al., 2020). While inclusion was initially focused on groups traditionally occupying a marginalized position in education (including students with disabilities, from ethnic minority descent or disadvantaged families), inclusive education is currently conceptualised as a call to transform educational systems at large to reach all students regardless of their background (Opertti et al., 2014). This perspective on inclusion is also known as Education for All (EfA) (Ainscow & Miles, 2008).

EfA can be framed as a so-called wicked problem (Armstrong, 2017), being a social problem that is both hard to grasp and solve. Wicked problems are characterised by institutional complexity requiring cooperation and changes to several processes, while scientific knowledge on the topic is often fragmented or contested (Crowley & Head, 2017; Rittel & Webber, 1973). While effective EfA would require changes to curricula, teacher behaviour, and cooperation between educational stakeholders (i.e., institutional complexity) (Dyson et al., 2002), most research on EfA employs a narrow focus by considering only one specific diversity group, and is either literature-based or qualitative in nature (Messiou, 2017). While this research has highlighted the crucial role of teachers and has given us some hints on how EfA can be realised, these have not been empirically tested on a large scale due to the lack of valid measurement instruments. Consequently, this article provides insight into the validation of the “DIversity SCreening in educatiOn" (DISCO)-instrument, which is an instrument designed to grasp teachers’ competences for inclusive education, and which could help both research and practice move forward in tackling EfA as a wicked problem.

Teachers’ competences in inclusive education

Inclusive teaching has become an obligatory professional competence for teachers in many countries (Burns & Shadoina-Gersing, 2010). Unfortunately, teachers both in Flanders and abroad express a low capacity to address the educational needs of diverse pupils (de Bruïne et al., 2014; Siongers et al., 2021). Hence, research that provides more insight on teachers’ competences for inclusion and the contexts in which these competences can flourish, is sorely needed. Teacher competence for inclusive education is defined as being a complex combination of knowledge, skills and attitudes that allow a teacher to successfully respond to pupils’ diversity in the classroom (Crick, 2008). However, it has to be recognized that developing instruments to grasp teachers’ inclusive competences is not an easy task, due to

the complex nature of the concept of inclusive education, which entails both a broad scope (i.e., not being focused on a specific group but rather transcending any diversity group), and shows institutional complexity by requiring several educational processes at once to realize maximal learning- and developmental outcomes for students (Mitchell, 2007). Furthermore, the concept of competence an sich has been extensively debated as well. While some researchers have defined competence as actual behaviour in real-world situations, it has been suggested that more can be learned by considering competences as a process which results in observable behaviour while simultaneously acknowledging underlying factors such as knowledge and motivation that underpin and affect this behaviour. Hence, Blömeke et al. (2015) propose to view competences along a continuum with dispositions (such as knowledge and beliefs) and performance being connected through situation-specific skills. These situation-specific skills then entail processes such as perception and decision-making, which determine in a specific situation how dispositions are translated into performance (Blömeke et al., 2015; Santagata & Yeh, 2016). As such, these situation-specific skills are akin to the concept of self-efficacy from social cognitive theory, which proposes that human achievement is dependent on one's behaviours, internal personal factors (such as cognitions and beliefs) and environmental conditions (Bandura, 1997; Schunk & Pajares, 2001). Consequently, in order to get a broad image of aspects central to teachers competences for inclusive education, research is needed that combines an investigation of teachers' beliefs on diverse learners (i.e., the scope of inclusive education) and simultaneously, teachers' assessment of their capabilities to realize certain outcomes for students, that is self-efficacy. Both aspects, being beliefs on diversity and sense of self-efficacy, will be discussed in detail below.

Teachers' beliefs

With regard to teachers' beliefs on diverse learners, two distinct but related aspects are

central, being teachers' professional beliefs on student diversity and teachers' beliefs about the responsibility of education towards diverse learners. With regard to the latter, teachers' view on whether equity is part of the central aims of education and hence, what constitutes their responsibilities as a teacher, has a profound impact on shaping their classroom behaviour (Lauermann, 2013). However, teachers tend to report more negative attitudes towards inclusive education than either pupils or parents (Van Mieghem et al., 2020), while a qualitative study shows that teachers who consider it to be the responsibility of education to achieve both excellence and equal learning opportunities, tend to devote themselves more completely to realizing quality classes and supporting struggling students from diverse backgrounds (Fischman et al., 2006). In short, the degree in which teachers consider providing EfA as one of their central responsibilities, will be central in shaping their willingness to invest time and effort in the creation of inclusive classrooms. Interestingly, responsibility of teachers should not only be assessed with regard to learners, but also regarding colleagues and parents (Lauermann & Karabenick, 2011). Research suggests that schools with strong parent-teacher connections are better at realizing maximal developmental chances for their students (Dyson et al., 2002), and that achieving equitable learning environments requires a shared responsibility and collaboration among teachers (Killoran et al., 2014). Consequently, in order to really grasp teachers' competences in creating inclusive education, collaboration with these partners should not be disregarded.

Besides this view on the goals of education (without which a teacher would have no incentive to realize inclusive education), teachers' beliefs on learners from diverse backgrounds is of central importance as well. In line with the work of Pohan and Aguilar (2001), these are called teachers' professional beliefs about diversity. These beliefs subconsciously influence teacher behaviour, for instance by involving students for whom teacher have lower expectations less actively in classroom activities or by seeing poor

performance as normal (Jussim & Harber, 2005; Murdock-Perriera & Sedlacek, 2018). This process has a negative impact on both students' cognitive outcomes and well-being (Demanet & Van Houtte, 2012). Importantly, such effects of teacher expectations tend to be especially powerful for at-risk students, such as students from lower socioeconomic backgrounds and ethnic minority descent (Jussim & Harber, 2005). Hence, in the framework of inclusive education, it is particularly important to understand and assess teachers' beliefs on teaching diverse learners, to counter the negative self-fulfilling prophecies for these students. Unfortunately, research on teachers' beliefs towards diverse learners often employs a narrow definition of diversity, for instance by focusing only on teachers' multicultural beliefs (e.g., Hachfeld et al., 2015), or beliefs on students with behavioural difficulties (e.g., MacFarlane & Woolfson, 2013). While such specific beliefs allow for a fine-grained analysis of educational barriers for particular student groups, it remains unclear how teachers' beliefs towards several groups of students relate to each other and whether an overall inclusive mindset among teachers exists. Consequently, to fundamentally advance the research on classrooms that are effective for all students, research must consider teacher beliefs towards several groups of students simultaneously.

Hence, we propose to assess teachers' beliefs on diverse learners by assessing 1) teachers' professional beliefs towards students along important axes of diversity (i.e., ethnicity, disability, gender & sexual orientation, and socio-economic status) and 2) teachers' beliefs on educational responsibility with regards to diverse students, parents and colleagues.

Teachers' sense of efficacy

Besides teachers' beliefs on diverse learners, the capability to create the educational preconditions necessary for students' maximal learning is fundamental. People's beliefs in their own capability to perform certain tasks is called self-efficacy (Bandura, 1997) and self-

efficacy beliefs have proven to be powerful predictors of teachers' motivation and actual performance (Klassen et al., 2011). A high sense of self-efficacy is especially powerful in the face of adversity, in order to persevere when faced with difficulties or failure (Bandura, 1997). Since realizing inclusive learning environments requires a constant effort on the part of teachers to continuously monitor, evaluate and adapt their teaching methods to best suit the needs of the students present (Giangreco et al., 1995), self-efficacy could constitute a powerful resource. Importantly, research has shown that self-efficacy is not unidimensional, but tends to be task-specific (Huang, 2013). Consequently, it is important to consider teachers' self-efficacy with regards to several necessary tasks to create inclusive classroom environments rather than one overall assessment. Hence, in this research, teachers' self-efficacy beliefs with respect to several central aspects of realizing inclusive learning environments will be assessed. That is: 1) noticing diversity, 2) creating powerful learning environments for diverse students, and 3) realizing a positive classroom climate for diverse students. The first subcomponent of noticing diversity, taps the extent to which teachers see themselves capable of actively gathering information on several aspects of student diversity (f.i., students' background, interests, well-being, …). Only when teachers are aware of and exploit these differences, can a classroom environment be maximally adapted to the strengths and needs of every student (Tomlinson et al., 2003). In tandem with this noticing, the second subcomponent taps the extent to which teachers feel capable to design a classroom environment that takes into account the strengths and needs of their students. The third subcomponent measures the extent to which teachers feel capable to create positive classroom climates for their students, since research has shown both the impact teachers have on the creation of qualitative relationships among students (Wellborn et al., 1992) as well as the vital importance of this for at-risk students' well-being and achievement (Hamre & Pianta, 2001).

As discussed above, there is currently a lack of valid instruments to grasp teachers' competence for inclusive education, due to the institutional complexity and broad scope of EfA. That is, to truly capture competence for EfA, researchers requires an instrument that considers both teachers *willingness* to work on inclusion (i.e., beliefs) as well as their *capabilities* to create inclusive learning environment (i.e., self-efficacy). Furthermore, existing instruments tend to focus on either a specific teaching sub-component, such as class management or differentiated instruction, or on a specific student group, such as students with a disability or ethnic minority background, while EfA transcends specific diversity groups and encompasses several educational processes necessary for maximal learning outcomes. Hence, the goal of this article is to establish a valid and reliable instrument to measure teachers' beliefs and self-efficacy with regards to inclusive education. Furthermore, teachers' scale-scores on this validated instrument are analysed in order to gain insight on teachers' thinking about diversity in the classroom.

Methodology

Sample & respondents

In order to allow for a process of trial and improvement as well as cross-validation, data was collected in two phases. In the first phase, data was collected in the fall of 2017 in a sample of teacher education institutions. All teacher education institutions within a Belgian province were invited to participate. Seven institutions decided to participate in the study, translating to a total number of 975 pre-service teachers filling out the online research instruments. Of these, 167 students were in training to become a nursery teacher, 155 were studying to become primary school teachers, whereas 143 and 433 were studying to become respectively lower or higher secondary school teachers. 75% of the sample were women, while the mean age was 27.38 with a range from 17 to 57. By the end of the survey, 647 students had

provided valid answers, translating to an attrition rate of 33.6%.

In the second phase, data was collected with in-service teachers in the spring of 2018. All schools within a Belgian city were invited to participate. Of the 40 contacted schools, 28 participated in the study, translating to a participation rate of 70%. All teachers within each school were invited to fill out the online survey (N = 600). Of these, 210 were working in a primary school, while 384 were situated in a secondary school. 73.2% of the sample were women, while the mean age was 41.02 with a range from 22 to 63. When comparing the data to previous descriptions of the Flemish teaching population (Deneire et al., 2013; Groenez et al., 2018), we see a congruence for both age and gender divisions, indicating that no systematic biases seem to have occurred. By the end of the survey, 509 respondents provided valid answers, translating to an attrition rate of 15.2%.

Due to the anonymous and non-invasive nature of the research (being a voluntary, one-time participation of adults in survey-research on professional practice), no official approval by the ethics committee was sought. Following the European Code of Conduct for Research Integrity, all respondents agreed to a written informed consent stating that participation in the research was voluntary, that they could stop their participation at any moment, and that their answers would be processed anonymously.

Instrument

Item creation and selection

First, a pool of items was created by three researchers with expert knowledge on diversity in education. This brainstorm was based on an existing reflection-instrument for how teachers address diversity in the classroom, and validated scales on ethnocentrism, homonegativity, differentiation, collaboration, and teaching efficacy. During this creation phase, the following criteria were used: (1) items had to either refer to a specific diversity group or be applicable to

all children; (2) items needed to explicitly measure a single practice or perception; and (3) items needed to be relevant across multiple teaching contexts. In order to ensure content validity and readability, these items were then adapted based on feedback by two academics in the field of diversity and (teacher) education, two in-service teacher educators specialized in teacher professional development for diversity, and two teachers. Through this process, the questionnaire ended up consisting of 125 items, which were then presented in the first phase of the research to a large-scale group of pre-service teachers. The questionnaire was administered in Dutch.

The questionnaire

The questionnaire consists of two parts. Part one contains 42 items considering self-efficacy, consisting of several dimensions. In line with other validated efficacy scales (Mone et al., 1995; Tschannen-Moran & Hoy, 2001), these items are preceded by the stem "To what extent are you able to…" and is answered on a 9-point Likert scale (0 = *not at all*, 8 = *a lot*). The first dimension contains 9 items tapping teachers' perceptions of being able to notice student diversity (f.i., students' background, interests, well-being, …) with, for instance, "make time for in-depth individual conversations with all of your students in order to provide more effective support". The second dimension taps the extent to which teachers feel capable to design a classroom environment that takes into account the needs of their students and contains 21 items (e.g., "Give students opportunities beyond traditional tests and exams to demonstrate their knowledge and skills"). The third dimension measures the extent to which teachers feel capable to create positive interactions among students through 12 items (e.g., "guide students in dealing with other opinions in a respectful way").

The second part of the questionnaire considers teachers' beliefs by answering on a 7-point Likert scale (0 = *completely disagree*, 6 = *completely agree*) to allow for nuanced

answers, in line with psychometric recommendations for Likert-scales (Joshi et al., 2015). The first component taps teachers' professional beliefs on diversity, specifically with regards to students with ethnic minority background (e.g., "The more immigrant students in a school, the more discipline problems the school will face"), with a disability (e.g., "Students with a disability reduce the learning opportunities for the other students in the class"), from lower socio-economic backgrounds (e.g., "underprivileged parents are often less interested in their children's progress in school than other parents"), with regards to gender (e.g., "I think it's good that girls are not asked to help out with jobs in school that involve heavy lifting"), and LBGT-students (e.g., "many students experiment with their sexuality just to get noticed").

The second component consists of responsibility-beliefs, and is divided in two sections. The first one taps teachers' perceptions of to what extent they should collaborate with both diverse parents and colleagues. These 13 items are preceded by the stem "It is my job to…" followed by, for instance, "Establish a good relationship with all parents, even if I do not like them" or "learn from colleagues with a completely different teaching approach". The second section taps the extent to which teachers believe it is the responsibility of educational professionals to create maximal developmental opportunities for diverse students. These 42 items consider general items (e.g., "teachers have a pedagogical responsibility in the classroom, not of upbringing or support") as well as with regards to specific groups of students (e.g., "The school must allow students with a migration background to speak a language other than Dutch at school", "it is time to pay attention to LGB-sexuality in several parts of the curriculum").

Plan of analysis

To validate the factor structure underlying the DISCO-instrument, we combined exploratory (EFA) and confirmatory factor analyses (CFA) in a stepwise approach. In the first step,

exploratory factor analysis using SPSS 25 was chosen to identify a (set of) latent constructs underlying the measured items without strong theoretical assumptions on how many factors existed (Field, 2009). Because teachers' competences in handling diversity were expected to correlate (Field, 2009; Roose et al., 2018), Principal Component Analysis (PCA) with oblique rotation technique (direct oblimin) was used. The assumptions to perform EFA were checked: data distributions were checked to ensure that variables were roughly normally distributed (Field, 2009) and the correlation-matrix was inspected to identify scale items that correlated below |.30| with all other variables or above |.80|. The suitability of the data for the exploratory analysis were checked by considering the Kaiser–Meyer–Olkin test (KMO) which should exceed the recommended value of .60, and the Bartlett's test of sphericity which should be significant. Furthermore, the anti-image correlation matrix was checked for negative correlations, low communalities or high partial correlations (Field, 2009). To determine the number of factors, both the scree plots' point of inflexion and Kaiser's criterion (eigenvalue >1) were checked (Field, 2009). Items were eliminated if they had inadequate (partial) correlations or high cross-loadings (>|.40|), if their variance was not well explained as suggested by low communalities, or if the loading on their designated factor was weak (<|.35|). Factors with a minimum of three items and Cronbach's alpha >.70 were retained for the second step (Field, 2009).

In the second step, the initial factor solution from step 1 was checked with in-service teachers using confirmatory factor analyses in Mplus. This technique allows us to check to what extent a hypothesized model fits the data. An MLR estimation was used, and goodness of fit indicators were checked: CFI values larger than 0.95, X^2/DF smaller than two, RMSEA values smaller than 0.05 and SRMR values smaller than 0.05 are considered good (Schermelleh-Engel et al., 2003). Where necessary, the model was optimized based on suggestions of the modification indices, standardized factor loadings, R-square estimates of

the items, and theoretical interpretability of the factor. To compare models, we checked which model had a lower AIC value. Note that X^2 statistics are reported, but not discussed because the test is sensitive to large sample sizes (Kline, 2011).

Finally, based on these results, the scale scores were obtained by computing a mean for each respondent with a maximum of 25% missing values on the scale. The final set of items for each scale can be seen in Table 1 and 2. Repeated measures analysis of variance with a bonferroni post-hoc test was conducted to examine possible significant differences on scale scores for efficacy and beliefs.

Results

Step 1: EFA with pre-service teachers

Self-efficacy in noticing student diversity

The 9 items had a KMO value of .902 and Bartletts' test was significant ($X^2 = 4584$, df = 36, $p < .001$). Inspecting the anti-image correlation matrix led to the deletion of three items. PCA on the remaining 6 items revealed satisfactory KMO-value, anti-image correlations and Bartletts' test. The scree plot and Kaiser criterion indicated one factor to extract with an eigenvalue 4.00, which explained 67% of the total variance. Factor loadings ranged from .76 to .84, and communalities ranged from .58 to .72. The scale had high internal reliability (α = .90).

Self-efficacy in creating high-quality student-interactions

Based on the data distributions and anti-image correlation matrix, four items were deleted. PCA on the remaining 8 items revealed satisfactory KMO-value, anti-image correlations and Bartletts' test. The scree plot and Kaiser criterion indicated one factor to extract with an eigenvalue 5.18, which explained 64.7% of the total variance. Factor loadings ranged from

.70 to .85, and communalities ranged from .49 to .72. The scale had high internal reliability (α = .92).

Self-efficacy in creating stimulating learning environments for diverse students

Inspection of the correlation matrix and anti-image correlation matrix revealed several problematic item-sets, so eight items were deleted. PCA on the remaining 13 items revealed satisfactory KMO-value, anti-image correlations and Bartletts' test. The scree plot and Kaiser criterion indicated one factor to extract with an eigenvalue 7.43, which explained 57.2% of the total variance. Factor loadings ranged from .70 to .80, and communalities ranged from .48 to .63. The scale had high internal reliability (α = .94).

Professional beliefs about diverse students

Based on the data distributions, correlation matrix and anti-image correlation matrix, ten items were deleted. PCA on the remaining 27 items revealed satisfactory KMO-value, anti-image correlations and Bartletts' test. The scree plot and Kaiser criterion indicated that it was reasonable to retain four factors. Inspection of the rotated solution revealed an item loading below .3 unto its designated factor, which was deleted. The PCA on the remaining 26 items similarly revealed that it was reasonable to retain four factors. Communalities ranged from .32 to .60. These four factors explained 46.48% of the total variance and had eigenvalues of 25.81, 9.83, 6.13, and 4.71 respectively. To interpret these factors, we made use of an oblimin rotation. The factor correlation matrix indeed shows that there was a moderate correlation between the various factors (r-values between |.152| and |.363|). The first factor consisted of 6 items tapping beliefs towards ethnic minority students, with factor loadings ranging from .38 to .77, and a Cronbach's alpha of .77. The second factor consisted of 9 items tapping beliefs towards gender and LGBT-students, with factor loadings ranging from .51 to .69, and a Cronbach's alpha of .82. The third factor consisted of 6 items tapping beliefs towards students

with a disability, with factor loadings ranging from .57 to .72, and a Cronbach's alpha of .77. The final factor consisted of 5 items tapping beliefs towards low SES-students, with factor loadings ranging from .42 to .72, and a Cronbach's alpha of .72.

Responsibility of the educational field to create Education for All

Based on the data distributions, correlation matrix, and anti-image correlation matrix, fifteen items were deleted. PCA on the remaining 18 items revealed satisfactory KMO-value, anti-image correlations and Bartletts' test, and that it was reasonable to retain four factors. Communalities ranged from .41 to .72. These four factors explained 53.98% of the total variance and had eigenvalues of 25.42, 11.55, 9.04, and 7.97 respectively. The factor correlation matrix shows that there was a correlation between the various factors (r-values between |.134| and |.281|). Interestingly, the revealed factors did not line up directly with the diversity groups included in the items. Rather, the first factor, consisting of 6 items, seemed to tap a broad sense of school responsibility, including general items and items with regards to tackling SES-based and gendered discrimination. This factor had loadings ranging from .62 to .75, and a Cronbach's alpha of .71. The second factor tapped beliefs on school approaches specifically directed at ethnic minority students, consisting of 4 items with loadings ranging from .45 to .86, and a Cronbach's alpha of .70. The third factor consisted of 4 items representing beliefs on the broad role teachers should take on, with factor loadings ranging from .59 to .71, and had a Cronbach's alpha of .61. The final factor consisted of 4 items tapping beliefs on schools' responsibilities towards students with a disability, with factor loadings ranging from .64 to .83, and had a Cronbach's alpha of .75.

In acknowledgement of this rather surprising factorial structure, we decided for the second phase to add a few more items representing a general sense of schools' responsibilities towards all diverse students, rather than geared towards specific groups. However, the items

pertaining to the factor representing teachers' broad role were dropped from the questionnaire for the second phase, due to its low Cronbach's alpha.

Responsibility beliefs towards diverse parents and colleagues

Inspection of the data distributions revealed that respondents had answered the items in a rather extreme manner. That is, the median-likert category was at least 5 = *agree* for all items, indicating that 50% of respondents scored 5 or higher on scales going from 0 to 6. As this indicated severe ceiling-effects, it was decided to rework the dimension as a whole before submitting the questionnaire to in-service teachers. It was hypothesized that the stem "It is my job to…" was leading to a certain social desirability bias rather than providing a reflection of actual intentions. Consequently, it was deemed advisable to rework the scale to tap respondents self-efficacy in dealing with diverse parents and colleagues. Hence, in-service teachers were presented with 16 items preceded by the stem "To what extent are you able to…" and a 9-point Likert scale (0 = *not at all*, 8 = *a lot*).

Step 2: CFA with in-service teachers

In the second step, the solution that resulted from the first step was checked for in-service teachers using CFA. With regards to self-efficacy, we included four different factors being noticing diversity with six items, enabling high-quality student-interactions with eight items, creating stimulating learning environments with 13 items, and collaborating with colleagues and parents with 16 items. With regard to beliefs about diverse students, we included four factors geared toward specific groups, being ethnic minority students, disabled students, students from low SES and gendered issues with respectively six, six, nine, and five items. For beliefs on the responsibility of the educational field to create education for all, we included three factors focusing on general school policy, initiatives geared specifically towards either ethnic minority students or students with a disability, with respectively eight,

four and four items. The result of this points to acceptable but not good fit indices (AIC = 155650, X^2= 8030, DF = 3430, $p < 0.001$, X^2/DF = 2.34, RMSEA = 0.048, CFI = 0.760, SRMR = 0.066). Changes were made in order to achieve a better fit. As factor loadings for the items related to collaborating with colleagues were consistently lower (i.e., between .424 and .511), we tested a model with a separate factor pertaining to collaborating with diverse parents (consisting of 10 items) and one pertaining to collaborating with colleagues (consisting of five items). These adaptations led to a better fit (AIC = 154671, X^2= 7125, DF = 3419, $p < 0.001$, X^2/DF = 2.08, RMSEA = 0.043, CFI = 0.807, SRMR = 0.059). In a next step, 13 items were deleted as suggested by the modification indices, R-square values or factor loadings. This led to a further improved model (AIC = 133214, X^2= 4765, DF = 2418, $p < 0.001$, X^2/DF = 1.97, RMSEA = 0.041, CFI = 0.846, SRMR = 0.054). In accordance with suggestions by Hu and Bentler (1999) and as shown by Coubergs and colleagues (2017), we accept a combination of CFI-values approaching .90 with RMSEA values smaller than .06 and SRMR-values under .08 as good model fit. Factor loadings can be seen in Table 1 and 2 (note that the loadings for self-efficacy and beliefs are shown in separate tables for lay-out reasons only, as these are tested as one model within CFA). This table shows loadings above |.50| for all items, with the exception of C2_14, C2_27, C2_29, C5_14, and C5_15. As these items still loaded above |.40|, while deleting them was not conducive to the Cronbach's alpha of the respective factor, this was deemed acceptable. All in all, this results in a model with five factors related to self-efficacy and seven factors related to beliefs, with alpha scores being good for each factor and showing that no items could be deleted in order to get a higher alpha. More specifically, the factors related to self-efficacy comprises "noticing student diversity" consisting of 6 items with α = .87, "enabling high-quality student-interactions" consisting of 6 items with α = .85, "creating stimulating learning environments" consisting of 13 items with α = .90, "collaborating with colleagues" consisting of 3 items with α = .83, and "collaborating

with parents from diverse backgrounds” consisting of 8 items with α = .88. The factors relating to beliefs comprises professional beliefs on different types of diversity, specifically on ethnic minorities (# of items = 6, α = .79), students with a disability (# = 4, α = .78), people from disadvantaged families (# = 5, α = .76), and gender (# = 8, α = .79), as well as beliefs on the responsibility of the educational field to create education for all, including a factor on general school policy (#= 5, α = .74), and factors with initiatives geared specifically towards ethnic minority students (#= 4, α = .70), and students with a disability (# = 4, α = .73).

Scale-scores

As shown in Table 3, teachers tended to score on average between 5 and 6 on the efficacy-scales ranging from 0 to 8, indicating that teachers feel more or less “reasonably” efficacious. Analysis of variance showed that there were significant differences on efficacy scores depending on the dimension (V = .438, F(4, 520) = 101.26, p < .001). The post-hoc Bonferroni test revealed that teachers did not differ significantly in their efficacy in enabling high-quality interactions between students and collaborating with colleagues. These were the dimensions on which teachers tended to feel most efficacious when compared to the other dimensions (p < .001), with creating stimulating learning environments taking a middle position (p < .01), while noticing student diversity and collaborating with parents did not differ significantly from each other and were the dimensions that teachers rated themselves least efficacious on (p < .01).

With regard to beliefs, analysis of variance showed that there were significant differences on belief scores depending on the dimension (V = .825, F(6, 514) = 101.26, p < .001). More specifically, teachers’ scores varied on average between 3 and 5 on scales ranging from 0 to 6, indicating that teachers’ beliefs varied from a neutral position to “agree” depending on the specific scale. The post-hoc Bonferroni test revealed that, with the exception

of teachers' beliefs on SES students and their beliefs on school policy specifically geared towards disability students ($p < .887$), all scale scores differed significantly from each other. Hence, teachers scored highest on their belief on schools' responsibility to have a general policy on diversity, followed by holding most favourable beliefs with regards to students' gender, and students with a disability, followed by ethnic minority students. Teachers held the least positive beliefs on the responsibility of a school to have a policy specifically geared towards ethnic minority students, and scored somewhat higher on their beliefs on SES students and the responsibility of a school to have a policy specifically geared towards disability, which did not differ significantly (as reported above).

Discussion

While inclusive education has achieved international importance as a leading educational movement (Burns & Shadoina-Gersing, 2010), it can be considered a wicked problem as it is characterised by normative plurality, institutional complexity and scientific insecurity (Armstrong, 2017; Rittel & Webber, 1973). This article aims to help grasp the issue by validating the DISCO-instrument, which measures teachers' beliefs and sense of efficacy towards diversity in education. In what follows, we discuss not only the factorial structure arising from the data, but also explore what this unveils on teachers' thinking about diversity in the classroom.

The questionnaire consists of two parts: beliefs and sense of efficacy. Teacher beliefs consist of 36 items in total, and includes beliefs about student diversity and beliefs about the responsibility of education to create education for all. The beliefs on student diversity consist of several dimensions, specifically teachers' professional beliefs about students with an ethnic minority background, with a disability, from lower socio-economic backgrounds, and beliefs

about students' gender. While these dimensions match with typically delineated categories of at-risk students, this was not entirely the case for gender and sexual orientation. Interestingly, even though items on both LGBT students and gender norms were included, analyses show that both pre- and in-service teachers tend to think of these as one overarching construct. This is in line with the theoretical concept of heteronormativity, which highlights the way in which normative ideas on gender expression and sexuality are interwoven (Habarth, 2015). Hence, the validation of the DISCO-instrument lends further support to the construct of heteronormativity, not just from a theoretical perspective but within the minds of teachers as well.

The responsibility-items tap the extent to which teachers believe it is the responsibility of educational professionals to create maximal developmental opportunities for diverse students. This includes several dimensions, including one about general school policy, which encompasses both general items (e.g., "It is every teacher's job to contribute to a school policy that is mindful of diversity") as well as items referring to SES- and gender-issues (e.g., "Students from families with financial problems should be provided with appropriate support (e.g. payment plan, special support fund)", "As a school, it is important to break the traditional divide between subjects that are stereotypically for boys or for girls."). Other dimensions encompass items on school policy directed towards specific diversity-groups, with one dimension specifically geared towards students with a migration background, and another geared towards students with a disability. This factorial structure suggests some interesting things about the way teachers think about school policy and the educational field's responsibility on EfA. For instance, issues regarding SES and gender are apparently being regarded as "commonplace enough" to be integrated within general school policy. Conversely, the needs of ethnic minority students and those with a disability are seen as being "too different" and warrant their own specific policies and guidelines. Previous research has

shown that Flemish teachers tend to have a lot of questions on how to handle students' multilingualism and religiosity (Groenez et al., 2018). Furthermore, Flanders historically has a high rate of students segregated into special education when compared to other European nations (Ruijs & Peetsma, 2009), and has only recently made substantial steps in integrating students with a disability in mainstream education (Groenez et al., 2018). Hence, it stands to reason that this is contributing to teachers seeing policies for both ethnic minority students and those with a disability as being "special". It would be interesting for future research to explore to what extent teachers in other contexts share a similar view. Hence, we call for a cross-national validation of the DISCO-instrument

When taking a closer look at how teachers score on belief-scales, some interesting findings emerge. First, teachers tend to score highest on the responsibility of schools to contribute to general school policies for diversity, and have positive beliefs about students' gender. The positive stance of Flemish teachers with regard to gender norms and LGBT-rights is in line with previous research (Groenez et al., 2018), and matches the overall progressive stance of Belgium when it comes to LGBT-rights (ILGA-Europe, 2020). On the other side of the spectrum, Flemish teachers tend to think least positive about school policies specifically geared toward ethnic minority students and those with a disability, as well as having less positive beliefs about SES students. Noteworthy is how teachers combine positive views on general school policies (which include items on providing for low-SES students) with less approving personal beliefs on SES-students. Perhaps this shows that teachers fully agree with the uplifting role that education could play and in which strong, general policies have a substantial role, while at the same time acknowledging the challenges of providing education on a day-to-day basis to students from vulnerable family backgrounds (Dyson et al., 2002). Qualitative research would be well-placed to thoroughly investigate and make sense of this ambiguous stance of teachers.

The second part of the questionnaire contains 36 items considering teachers' sense of efficacy, and is organized in five dimensions. The first dimension taps teachers' efficacy in noticing student diversity (f.i., students' background, interests, well-being, …), the second the extent to which teachers feel capable to design a classroom which capitalizes on the strengths and needs of their students, and the third measuring the extent to which teachers feel capable to create high-quality interactions among students. The fourth and fifth dimension tap teachers' perceptions of the degree to which they feel capable to collaborate with colleagues and diverse parents respectively.

The analyses on the scale-scores show that teachers tend to feel most efficacious in both collaborating with colleagues and in creating high-quality interactions among students. This is followed by creating stimulating learning environments for diverse students. Note that this dimension includes a lot of items related to providing differentiated instruction, with using a variety of instructional and evaluation methods, and stimulating the interest and engagement of students. As such, this dimension connects to the core of teachers didactical competencies (Dixon et al., 2014; Tomlinson & Moon, 2013). Consequently, the ranking of these dimensions may come as no surprise, with teachers feeling reasonably efficacious in this dimension, while at the same time continuing to see opportunities for further growth and improvement. At the same time, noticing student diversity and collaborating with diverse parents are the lowest rated efficacy-dimensions. Nevertheless, in order to capture students' attention and engagement, theories on differentiated instruction emphasize the importance of taking into account the social environment of students and their individual interests (Tomlinson & Moon, 2013). We could speculate that the relatively low score on this dimension reflects the idea among teachers that this is not a must, but merely a nice extra, second to more concrete didactical approaches. Qualitative research with teachers would be

well-placed to explore the reasons for teachers' efficacy-assessment on this dimension. Furthermore, the low score on collaborating with diverse parents is in line with previous research in Flanders, indicating that parents' involvement is often only expected or even tolerated under specific circumstances, such as coming to the parent-teacher contact moment or helping out with the school fair (Dom, 2006). Nevertheless, research has shown that creating strong connections between the school and the home environment is conducive to students' school belonging and achievement (Hattie, 2008; Henderson & Mapp, 2002), and could be an important lever in creating quality education for all (Dyson et al., 2002).

Note that the scales on efficacy in collaboration with parents and colleagues originally started out as responsibility belief-scales, which had to be reworked due to ceiling-effects. Interestingly, the data shows that teachers combine highest efficacy-ratings in collaborating with colleagues with lowest efficacy-scores in collaborating with parents. This shows both the nuances and complexities in (student-)teachers' assessments of what they *can* and *should* do. That is, our validation process suggests that (student-)teachers are able to recognize that collaboration is something they should do, while simultaneously admitting to low abilities in collaborating with one partner and high scores in collaborating with another.

Limitations

While this instrument is able to grasp teachers' beliefs for inclusive education as well as a self-assessment of their skills, it remains to be seen how the DISCO-instrument connects to other aspects of competency such as knowledge and actual classroom behaviour. Furthermore, the validation-process in this study remains largely focused on structural validity, while it has been argued that a full validation-process should encompass a.o. the stability of the instrument, as well as concurrent, discriminant and criterion-related validity (Zumbo & Chan, 2014). Especially a cross-national comparison could give us some interesting insights into

how contexts, with their specific educational realities and policies, shape teachers' thinking and competences on inclusive education.

All in all, this article discusses the DISCO-instrument, which measures teachers' beliefs and efficacy for creating quality education for diverse students. In the context of EfA being considered a wicked problem, we suggest that DISCO cannot only help researchers to grasp the state of affairs of a specific school or educational system, but can support policy makers and principals in making evidence-based decisions on the professionalisation needs of their school teams.

References

Ainscow, M., & Miles, S. (2008). Making Education for All inclusive: where next? *Prospects, 38*(1), 15-34.

Armstrong, D. (2017). Wicked problems in special and inclusive education. *Journal of Research in Special Educational Needs, 17*(4), 229-236.

[Record #121 is using a reference type undefined in this output style.]

Banks, J., Cochran-Smith, M., Moll, L., Richert, A., Zeichner, K., LePage, P., Darling-Hammond, L., Duffy, H., & McDonald, M. (2007). Teaching diverse learners. In L. Darling-Hammond & J. Bransford (Eds.), *Preparing teachers for a changing world: What teachers should learn and be able to do* (pp. 232-274). John Wiley & Sons.

Blömeke, S., Gustafsson, J.-E., & Shavelson, R. J. (2015). Beyond dichotomies: Competence viewed as a continuum. *Zeitschrift für Psychologie*(223), 3-13.

Burns, T., & Shadoina-Gersing, V. (2010). *Educating teachers for diversity: meeting the challenge*. Paris: OECD.

Crick, R. D. (2008). Key Competencies for Education in a European Context: narratives of accountability or care. *European Educational Research Journal, 7*(3), 311-318.

Crowley, K., & Head, B. W. (2017). The enduring challenge of 'wicked problems': revisiting Rittel and Webber. *Policy sciences, 50*(4), 539-547.

de Bruïne, E. J., Willemse, T. M., D'Haem, J., Griswold, P., Vloeberghs, L., & Van Eynde, S. (2014). Preparing teacher candidates for family–school partnerships. *European Journal of Teacher Education, 37*(4), 409-425.

Demanet, J., & Van Houtte, M. (2012). Teachers' attitudes and students' opposition. School misconduct as a reaction to teachers' diminished effort and affect. *Teaching and Teacher Education, 28*(6), 860-869.

Deneire, A., Vanhoof, J., Faddar, J., & Van Petegem, P. (2013). *Denken, handelen en professionele ontwikkeling van Vlaamse leraren en schoolleiders. Eerste resultaten van de Teaching and Learning International Survey (TALIS) [Thinking, acting and professional development of Flemish teachers and school leaders. First results of the Teaching and Learning International Survey (TALIS)]*. Ministry of Education and Training.

Dixon, F. A., Yssel, N., McConnell, J. M., & Hardin, T. (2014). Differentiated instruction, professional development, and teacher efficacy. *Journal for the Education of the Gifted, 37*(2), 111-127.

Dom, L. (2006). *Ouders en scholen*. Academia Press.

Dyson, A., Howes, A., & Roberts, B. (2002). A systematic review of the effectiveness of school-level action for promoting participation by all students (EPPI-Centre Review). *Research Evidence in Education Library. Issue 1.:*.

Field, A. (2009). *Discovering Statistics Using SPSS, Third Edition*. sage publications.

Fischman, W., DiBara, J. A., & Gardner, H. (2006). Creating good education against the odds. *Cambridge Journal of Education, 36*(3), 383-398.

Forghani-Arani, N., Cerna, L., & Bannon, M. (2019). The lives of teachers in diverse classrooms. *OECD Education Working Papers, 198*. https://doi.org/https://doi.org/10.1787/8c26fee5-en

Giangreco, M. F., J. Baumgart, D. M., & Doyle, M. B. (1995). How inclusion can facilitate teaching and learning. *Intervention in School and Clinic, 30*(5), 273-278.

Groenez, S., Vantieghem, W., Lamberts, M., & Van Avermaet, P. (2018). Diversiteitsbarometer Onderwijs Vlaamse Gemeenschap. In E. Keytsman (Ed.), *Diversiteitsbarometer onderwijs* (pp. 47–193). Unia.

Habarth, J. M. (2015). Development of the heteronormative attitudes and beliefs scale. *Psychology & Sexuality, 6*(2), 166-188.

Hachfeld, A., Hahn, A., Schroeder, S., Anders, Y., & Kunter, M. (2015). Should teachers be colorblind? How multicultural and egalitarian beliefs differentially relate to aspects of teachers' professional competence for teaching in diverse classrooms. *Teaching and Teacher Education, 48*, 44-55.

Hamre, B. K., & Pianta, R. C. (2001). Early teacher–child relationships and the trajectory of children's school outcomes through eighth grade. *Child Development, 72*(2), 625-638.

Hattie, J. (2008). *Visible learning: A synthesis of over 800 meta-analyses relating to achievement*. Routledge.

Henderson, A. T., & Mapp, K. L. (2002). *A new wave of evidence*. National Center for Family & Community: Connections with Schools.

Huang, C. (2013). Gender differences in academic self-efficacy: A meta-analysis. *European journal of psychology of education, 28*(1), 1-35. https://doi.org/10.1007/s10212-011-0097-y

ILGA-Europe. (2020). *Rainbow Europe 2020*. Retrieved 21/08/2020 from https://www.ilga-europe.org/rainboweurope/2020

Joshi, A., Kale, S., Chandel, S., & Pal, D. K. (2015). Likert scale: Explored and explained. *Current Journal of Applied Science and Technology*, 396-403.

Jussim, L., & Harber, K. D. (2005). Teacher expectations and self-fulfilling prophecies: Knowns and unknowns, resolved and unresolved controversies. *Personality and social psychology review, 9*(2), 131-155.

Killoran, I., Woronko, D., & Zaretsky, H. (2014). Exploring preservice teachers' attitudes towards inclusion. *International Journal of Inclusive Education, 18*(4), 427-442.

Klassen, R. M., Tze, V. M., Betts, S. M., & Gordon, K. A. (2011). Teacher efficacy research 1998–2009: Signs of progress or unfulfilled promise? *Educational Psychology Review, 23*(1), 21-43.

Kline, R. B. (2011). *Principles and practice of structural equation modeling, third*. New York: The Guilford Press.

Lauermann, F., & Karabenick, S. A. (2011). Taking teacher responsibility into account (ability): Explicating its multiple components and theoretical status. *Educational psychologist, 46*(2), 122-140.

Lauermann, F. V. (2013). *Teacher Responsibility: Its Meaning, Measure, and Educational Implications* University of Michigan].

MacFarlane, K., & Woolfson, L. M. (2013). Teacher attitudes and behavior toward the inclusion of children with social, emotional and behavioral difficulties in mainstream schools: An application of the theory of planned behavior. *Teaching and Teacher Education, 29*, 46-52.

Messiou, K. (2017). Research in the field of inclusive education: time for a rethink? *International Journal of Inclusive Education, 21*(2), 146-159.

Mitchell, D. (2007). *What really works in special and inclusive education: Using evidence-based teaching strategies*. Routledge.

Mone, M. A., Baker, D. D., & Jeffries, F. (1995). Predictive validity and time dependency of self-efficacy, self-esteem, personal goals, and academic performance. *Educational and Psychological Measurement, 55*(5), 716-727.

Murdock-Perriera, L. A., & Sedlacek, Q. C. (2018). Questioning Pygmalion in the twenty-first century: The formation, transmission, and attributional influence of teacher expectancies. *Social Psychology of Education, 21*(3), 691-707.

Opertti, R., Walker, Z., & Zhang, Y. (2014). Inclusive education: From targeting groups and schools to achieving quality education as the core of EFA. In L. Florian (Ed.), *The SAGE Handbook of Special Education* (pp. 149-169). Sage.

Pohan, C. A., & Aguilar, T. E. (2001). Measuring educators' beliefs about diversity in personal and professional contexts. *American educational research journal, 38*(1), 159-182.

Rittel, H. W., & Webber, M. M. (1973). Dilemmas in a general theory of planning. *Policy sciences, 4*(2), 155-169.

Roose, I., Goossens, M., Vanderlinde, R., Vantieghem, W., & Van Avermaet, P. (2018). Measuring professional vision of inclusive classrooms through video-based comparative judgement: An expert study. *Studies in Educational Evaluation, 56*, 71-84.

Ruijs, N. M., & Peetsma, T. T. D. (2009). Effects of inclusion on students with and without special educational needs reviewed. *Educational Research Review, 4*(2), 67-79.

Santagata, R., & Yeh, C. (2016). The role of perception, interpretation, and decision making in the development of beginning teachers' competence. *ZDM, 48*(1-2), 153-165.

Schermelleh-Engel, K., Moosbrugger, H., & Müller, H. (2003). Evaluating the fit of structural equation models: Tests of significance and descriptive goodness-of-fit measures. *Methods of psychological research online, 8*(2), 23-74.

Schunk, D., & Pajares, F. (2001). The development of academic self-efficacy. In A. Wigfield & J. Eccles (Eds.), *Development of achievement motivation.* (pp. 15-31). Academia Press.

Siongers, J., Spruyt, B., Van Droogenbroeck, F., Bongaerts, B., & Kavadias, D. (2021). *TALIS 2018 Vlaanderen-Verdiepend rapport diversiteit*. Vrije Universiteit Brussel.

Tomlinson, C. A., Brighton, C., Hertberg, H., Callahan, C. M., Moon, T. R., Brimijoin, K., Conover, L. A., & Reynolds, T. (2003). Differentiating instruction in response to student readiness, interest, and learning profile in academically diverse classrooms: A review of literature. *Journal for the Education of the Gifted, 27*(2-3), 119-145.

Tomlinson, C. A., & Moon, T. R. (2013). *Assessment and student success in a differentiated classroom*. ASCD.

Tschannen-Moran, M., & Hoy, A. W. (2001). Teacher efficacy: Capturing an elusive construct. *Teaching and Teacher Education, 17*(7), 783-805.

Van Mieghem, A., Verschueren, K., Petry, K., & Struyf, E. (2020). An analysis of research on inclusive education: a systematic search and meta review. *International Journal of Inclusive Education, 24*(6), 675-689.

Wellborn, J., Connell, J., Skinner, E., & Pierson, L. (1992). Teacher as social context (TASC). *Rochester, NY: University of Rochester*.

Zumbo, B. D., & Chan, E. K. (2014). *Validity and validation in social, behavioral, and health sciences*. Springer. https://doi.org/10.1007/978-3-319-07794-9

Table 1. Standardised CFA matrix for self-efficacy on handling diversity

To what extent are you able to ...?		Notice diversity	High-quality student-interactions	Stimulating learning environment	collaborate with parents	collaborate with colleagues
C1.02	Make time for in-depth individual conversations with all of your students in order to provide more effective support.	0.763				
C1.03	Gain insight into the learning needs of an individual student by consciously looking at how he/she responds to different tasks and works in different groupings.	0.686				
C1.05	Gain insight into a student's plans and dreams for the future.	0.674				
C1.06	Start a conversation with a student who is not attentive or is having a difficult time outside of the classroom.	0.688				
C1.07	Gain insight into social relationships among students.	0.775				
C1.09	Gain insight into the students' feelings about their family situation.	0.817				
C3.03	Allow students with learning difficulties to be helped by other pupils.		0.657			
C3.05	Give tasks where the students have to work together to complete the task successfully (for example, each having a different role).		0.619			
C3.06	Provide feedback on how students work together.		0.765			
C3.07	Create space in or outside the classroom to support students in the resolution of conflicts.		0.761			
C3.10	In group work, ensure that all pupils get to work with others from all across the class?		0.678			
C3.12	In the case of bullying, discuss a plan of action with the students involved.		0.710			

C4.01	Ensure that teaching materials reflect diversity in society.	0.580
C4.04	Use a wide range of evaluation methods in order to evaluate students in a broad a way as possible.	0.697
C4.06	Specifically check that students with a language delay have understood a question.	0.693
C4.07	Enter into dialogue with students about their results.	0.547
C4.08	Occasionally use a short moment of evaluation (e.g., quiz) to tailor your lesson in a more nuanced way to the students' needs.	0.693
C4.10	Give students opportunities beyond traditional tests and exams to demonstrate their knowledge and skills (e.g. through portfolios, writing tasks, oral presentations).	0.613
C4.11	Allow students to give input about content that they would like to cover in lessons.	0.685
C4.12	Have students evaluate your lessons.	0.537
C4.13	When managing the pace of your lessons, take account of students who work faster/slower than the others.	0.731
C4.14	Offer a variety of activities so that students can choose what to do themselves.	0.644
C4.16	Allow students to choose whether or not to use certain tools/support.	0.719
C4.18	Use a variety of teaching materials and media which invite pupils to draw on different senses.	0.686
C4.19	When necessary, adapt your learning objectives (knowledge and skills) to take account of differences between students	0.678

	(e.g., by creating main and advanced learning goals).		
C5.01	Make parents from different backgrounds feel comfortable about coming into school.	0.819	
C5.02	Help parents to support their children to do well in school.	0.870	
C5.03	Involve the parents of students with special needs in their children's educational career.	0.885	
C5.04	Adapt the way you communicate with different kinds of families (e.g. single parents, LGBT+ parents, non-native speakers…).	0.760	
C5.06	Use the insights that parents give you about their child.	0.706	
C5.07	Establish a good relationship with all parents, even if you do not like them.	0.720	
C5.14	Always be available for questions from parents during school hours.	0.480	
C5.15	Allow parents to give some input about my lessons.	0.451	
C5.11	Integrate lesson ideas from colleagues into your own teaching practice.		0.769
C5.12	Ask colleagues for feedback to improve your teaching.		0.801
C5.13	Create new teaching methods or materials together with colleagues in order to support students with learning difficulties.		0.800

Table 2. Standardised CFA matrix for beliefs in handling diversity

	To what extent do you agree with …?	Beliefs on different types of diversity				Schools' responsibility for EfA		
		Ethnic minorities	Gender	Disability	SES	Ethnic minorities	Disability	General policy
C2.01	Ethnic and cultural diversity is a valuable resource in education.	0.622						
C2.03	If there are more "native-born" pupils in a school, it means that academic achievement will be higher across the board.	0.451						
C2.04	If an additional language is spoken at home, the student will fall behind in their learning.	0.608						
C2.06	Immigrant students are not as good at mathematics as native students.	0.635						
C2.07	Students who speak a different language at home will never speak Dutch properly.	0.647						
C2.08	The more immigrant students in a school, the more discipline problems the school will face.	0.725						
C2.11	Many students experiment with their sexuality just to get noticed.		0.557					
C2.14	LGBT teachers (lesbian, gay, bisexual, transgender), have the right to be open with parents about their sexual orientation.		0.421					
C2.15	In order for their time in school to go smoothly, it is better for LGBT students not to openly display their sexual orientation.		0.709					
C2.16	I feel sorry for students who grow up in an LGBT family because it is not easy for them.		0.657					
C2.25	Boys shouldn't wear earrings or nail varnish when in school.		0.573					
C2.27	Two boys can dance together for the entire evening at the school dance without attracting undue attention.		0.477					
C2.28	I find it difficult to understand transgender students.		0.675					

C2.29	I think it's good that girls are not asked to help out with jobs in school that involve heavy lifting.	0.479				
C2.20	Students with a disability always score worse on tests than other students.		0.618			
C2.21	Students with a disability reduce the learning opportunities for the other students in the class.		0.681			
C2.22	Students with a disability often abuse the support that they receive.		0.756			
C2.24	I think that many teachers show too much understanding for the behaviour of students with a disability.		0.677			
C2.33	Underprivileged parents are often less interested in their children's progress in school than other parents.			0.669		
C2.34	It is difficult to be sympathetic towards poorer students if they or their parents always have the latest mobile phones or gadgets.			0.618		
C2.35	Schools that educate many underprivileged students have lower standards.			0.697		
C2.36	Underprivileged students rarely succeed in difficult subjects because they do not have supportive parents..			0.590		
C2.37	It is normal for teachers to have higher expectations of students from good backgrounds.			0.545		
C6.01	The school should provide specific services for students who want to follow religious practices (e.g., prayer room, halal/kosher food).				0.508	
C6.02	The school must allow students with a migration background to speak a language other than Dutch at school.				0.738	
C6.04	The school library should have books in the different home languages of the students.				0.676	
C6.05	I find it important to encourage students of immigrant origin to be proud of their ethnic and cultural background.				0.513	
C6.07	It is time that we give children with a disability a full place in mainstream education. It is their right.					0.765

C6.08	It is sensible to not send students to special educational support too quickly.	0.671	
C6.09	The school's infrastructure must be made accessible at all levels to people with physical and sensory disabilities (vision, hearing, motor).	0.548	
C6.10	You cannot expect the teacher to meet the needs of a child with a disability in mainstream education.	0.580	
C6.13	Teachers must be capable of working with students from different home situations.		0.608
C6.14	Students from families with financial problems should be provided with appropriate support (e.g. payment plan, special support fund for students who cannot pay the school fees).		0.688
C6.16	A teacher can make a difference for students from all kinds of home environment.		0.527
C6.21	As a school, it is important to break the traditional divide between subjects that are stereotypically for boys or for girls.		0.571
C6.44	It is every teacher’s job to contribute to a school policy that is mindful of diversity.		0.629

Table 3. Descriptive statistics of scale-scores

	Mean	SD	Range	N
**Efficacy**				
Noticing diversity	5,06	1,48	0-8	524
Enabling interactions	5,88	1,23	0-8	524
Creating learning environments	5,32	1,34	0-8	524
Collaborating with parents	5,09	1,55	0,13-8	524
Collaborating with colleagues	5,89	1,56	0-8	524
**Beliefs**				
Ethnic students	3,78	0,98	0,5-6	520
Students with a disability	4,20	1,00	1-6	520
Students' gender	4,83	0,85	1,75-6	520
SES students	3,38	1,10	0,6-6	520
General school policy	5,00	0,70	2,40-6	520
Policy geared towards ethnicity	2,93	1,21	0-6	520
Policy geared towards disability	3,49	1,12	0-6	520
